# 

**Published:** 1906-02-24

**Authors:** 


### NOTICE TO CORRESPONDENTS.

All MSS., Letters, Books for Review, and other matters intended for the Editor should be addressed The Editor, "The Hospital," The Hospital Buildings, 28 & 29 Southampton Street, Strand, W.C.

The Editor cannot undertake to return rejected MS., even when accompanied by stamped directed envelope.

**Notice to Advertisers and Subscribers.**

All Advertisements, Orders for Copies of The Hospital, and Business Communications should be addressed to **THE MANAGER (Not to the Editor),** The Hospital, 28 & 29 Southampton Street, Strand London, W.C.

The Cost of Subscription, post free, is as follows:—Per week, 3½d.; per quarter, 3s. 3d.; per half-year, 6s. 6d.; per annum, 13s. Foreign and Colonial:—Per annum, 19s. 6d., payable, in all cases, in advance.

**NOTICE.—Vol. XXXVIII. of "The Hospital" April to September 1905), in handsome cloth binding, will shortly be on sale at the office of this Journal, price 10s. 6d. Cases for binding the Numbers contained in this Volume 2s. Index to Vol. XXXVIII., 3½d., post free.**

**The Monthly Part for February is now ready. Price 1s., by post, 1s. 3d.**

### MEDICAL APPOINTMENTS AND VACANCIES.

### APPOINTMENTS.

BRISTOL ROYAL INFIRMARY.—Nixon, John Alexander, M.B. Cantab), M.R.C.P., Assistant Physician.

### VACANCIES.

CAMBRIDGE, ADDENBROOKE'S HOSPITAL.—House Surgeon. Also Assistant House Surgeon.

DEVON COUNTY ASYLUM.—Third Assistant Medical Officer.

EVELINA HOSPITAL, Southwark Bridge Road, London, S.E.—Asistant House Surgeon.

FARRINGDON GENERAL DISPENSARY AND LYING-IN CHARITY, 17 Bartlett's Buildings, Holborn Circus, E.C.—Honorary Physician.

ITALIAN HOSPITAL, Queen Square, London, W.C.—Honorary Assistant Dental Surgeon.

LEEDS, HOSPITAL FOR WOMEN AND CHILDREN.—Honorary Assistant Surgeon.

LEEDS PUBLIC DISPENSARY.—Junior Resident Medical Officer.

LEICESTER INFIRMARY.—House Surgeon.

LONDON TEMPERANCE HOSPITAL.—Assistant Resident Medical Officer.

METROPOLITAN EAR, NOSE, AND THROAT HOSPITAL, Grafton Street, Fitzroy Square, W.—Three Clinical Assistants and an Anæsthetist.

PUBLIC DISPENSARY, 59 Stanhope Street, Clare Market.—Resident Medical Officer.

TORBAY HOSPITAL, Torquay.—House Surgeon.

TRURO, ROYAL CORNWALL INFIRMARY.—House Surgeon.

WARWICK COUNTY LUNATIC ASYLUM.—Assistant Medical Officer.

### A NURSING CAREER

### HOW TO BECOME A NURSE.

### The Nursing Profession. How and Where to Train.

By Sir HENRY BURDETT, K.C.B.

Price 2/- Net; 2/4 Post Free.

**THE NURSE'S PRONOUNCING DICTIONARY OF MEDICAL TERMS AND NURSING TREATMENT.**

By Honnor Morten.

Fifth Edition, Edited and Revised by Mary I. Burdett.

2/- post free.

**NURSING HINTS TO PROBATIONERS ON PRACTICAL WORK.**

By Annesley Voysey.

Cloth, Illustrated, 2/- net, 2/3 post free.

**PRACTICAL GUIDE TO SURGICAL BANDAGING AND DRESSINGS.**

By Wm. Johnson Smith, F.R.C.S.

Illustrated, 2/- post free.

**ELEMENTARY ANATOMY AND SURGERY FOR NURSES.**

By William McAdam Eccles, M.S.Lond., M.B., F.R.C.S., L.R.C.P.

Profusely Illustrated, 2/6 post free.

**NURSING: ITS THEORY AND PRACTICE.**

Being a complete Text-book of Medical, Surgical and Monthly Nursing.

By Percy G. Lewis, M.D., M.R.C.S., L.S.A.

Profusely Illustrated, 3/6 post free.

**ELEMENTARY PHYSIOLOGY FOR NURSES.**

By C. F. Marshall, M.D., B.Sc., F.R.C.S.

Illustrated, 2/- post free.

**SURGICAL WARD-WORK AND NURSING.**

By Alexander Miles, M.D.Edin., C.M., F.R.C.S.E.

Copiously Illustrated.

3/6 net, 3/10 post free.

**A HANDBOOK FOR NURSES.**

By J. K. Watson, M.D., M.B., C.M.

New and Revised Edition.

Profusely Illustrated, 5/- net, 5/4 post free.

**SURGICAL INSTRUMENTS AND APPLIANCES USED IN OPERATIONS.**

A Classified and Illustrated List, with Explanatory Notes.

By Harold Burrows, F.R.C.S.

1/6 net, 1/8 post free.

**OPHTHALMIC NURSING.**

By Sydney Stephenson, M.B., F.R.C.S.E.

Profusely Illustrated.

3/6 net, 3/10 post free.

**A COMPLETE HANDBOOK OF MIDWIFERY.**

By J. K. Watson, M.D. Edin.

Over 350 pp., Profusely Illustrated with 143 Diagrams.

6/- net, 6/4 post free.

**NURSING IN DISEASES OF THE THROAT, NOSE AND EAR.**

by P. Macleod Yearsley, F.R.C.S.Eng., M.R.C.S., L.R.C.P. Lond.,

Illustrated, 2/6 post free.

**The Scientific Press, Ltd.,**

☛ *Complete Catalogue, post free, on application.*

Publishers of Nursing Manuals, Case-Books, and Charts of all Descriptions.

28 & 29 SOUTHAMPTON STREET, STRAND, LONDON, W.C.

